# The range of attraction for light traps catching *Culicoides* biting midges (Diptera: Ceratopogonidae)

**DOI:** 10.1186/1756-3305-6-67

**Published:** 2013-03-15

**Authors:** Carsten Kirkeby, Kaare Græsbøll, Anders Stockmarr, Lasse E Christiansen, René Bødker

**Affiliations:** 1 National Veterinary Institute, Technical University of Denmark, Bülowsvej 27, Frederiksberg C, DK-1870, Denmark; 2 Department of Informatics and Mathematical Modelling, Technical University of Denmark, Asmussens Allé, DTU, Building 305, Lyngby, DK-2800, Denmark

**Keywords:** *Culicoides*, Range of attraction, Vector abundance, Light traps, Vector monitoring

## Abstract

**Background:**

*Culicoides* are vectors of e.g. bluetongue virus and Schmallenberg virus in northern Europe. Light trapping is an important tool for detecting the presence and quantifying the abundance of vectors in the field. Until now, few studies have investigated the range of attraction of light traps.

**Methods:**

Here we test a previously described mathematical model (Model I) and two novel models for the attraction of vectors to light traps (Model II and III). In Model I, *Culicoides* fly to the nearest trap from within a fixed range of attraction. In Model II *Culicoides* fly towards areas with greater light intensity, and in Model III *Culicoides* evaluate light sources in the field of view and fly towards the strongest. Model II and III incorporated the directionally dependent light field created around light traps with fluorescent light tubes. All three models were fitted to light trap collections obtained from two novel experimental setups in the field where traps were placed in different configurations.

**Results:**

Results showed that overlapping ranges of attraction of neighboring traps extended the shared range of attraction. Model I did not fit data from any of the experimental setups. Model II could only fit data from one of the setups, while Model III fitted data from both experimental setups.

**Conclusions:**

The model with the best fit, Model III, indicates that *Culicoides* continuously evaluate the light source direction and intensity. The maximum range of attraction of a single 4W CDC light trap was estimated to be approximately 15.25 meters. The attraction towards light traps is different from the attraction to host animals and thus light trap catches may not represent the vector species and numbers attracted to hosts.

## Background

Biting midges (Diptera: Ceratopogonidae: *Culicoides*) are vectors of e.g. Bluetongue virus [1] and the newly discovered Schmallenberg virus in northern europe [2]. Due to their crepuscular activity pattern, the standard trapping method is by (UV) light traps [3]. The vision of *Culicoides* and Ceratopogonids in general has not been studied well [4], although their phototactic behavior is of epidemiological importance. This behavior influences their response to light traps, which are widely used for determining the presence, abundance and phenology of *Culicoides* (e.g. [5-7]).

An optimal sampling strategy to estimate insect abundance must rely on knowledge of the area or range covered by a single trap. Many different terms have been used for this measure, and we prefer to use the ‘range of attraction’, describing the (maximum) distance at which insects are attracted to the trap. This term allows for a non-symmetrical attraction range around the trap which is relevant for traps equipped with light tubes. Few studies have attempted to estimate the range of attraction for light traps: Odetoyinbo [8] carried out a study where a trap was hung at different distances from an open window which mosquitoes passed through at night. The aim was to estimate the point where the trap caught more than a simultaneously operated independent trap. Here, the ‘effective range’ for a CDC mini light trap was estimated to be approximately 5m. Baker & Sadovy [9] put up 125W mercury vapor lamps at different distances from the release point of marked moths. By varying the distance from the release point to the lamps, they found that the response distance was within 3m. More recently, Truxa & Fiedler (2012) [10] carried out a study where marked moths were released at different distances to a UV-light trap. The catches showed that the ‘radius of attraction’ was up to 40m in 5-min intervals. For *Culicoides*, Rigot & Gilbert (2012) [11] compared 8W Onderstepoort light traps and how they competed with each other at different distances. Background fluctuations in the *Culicoides* abundance were monitored by an independent light trap. The analysis assumed a fixed radius of each trap, regardless of the distance to other traps. The ‘effective trap radius’ was found to be approximately 30 m when the traps were running for 30-min intervals. However, Venter *et al.* (2012) [12], only found a ‘range of attraction’ for *Culicoides* for the same trap type of between 2 and 4 meters. In that experiment, two traps were hung at varying distances to each other and the background fluctuations were monitored using an independent trap. The ‘range of attraction’ was then the distance at which the two traps began to catch less than the independent trap. Both of these last studies hypothesized that *Culicoides* are attracted to the nearest trap, and that the light from the trap is isotropic (uniform in all directions). In the present study we incorporated the directionally dependent (anisotropic) light created by the light tubes in the light traps into two novel models for the attraction of *Culicoides* to light traps.

We tested three different models for attraction of *Culicoides* to light traps: Model I where the range of attraction for each trap is isotropic and independent of the distance to adjacent traps and where *Culicoides* fly to the nearest trap; Model II where *Culicoides* fly towards the direction with highest light-intensity, simulating anisotropic light and where overlapping ranges of attraction create an extended range of attraction; and Model III where overlapping ranges of attraction also create an extended range of attraction but where *Culicoides* continuously evaluate each light source in its field of view and fly towards the highest light-intensity. We used two different experimental setups to test the models. By fitting the models to the relative trap catches in the experimental setups we exclude factors influencing the level of abundance. The range of attraction for *Culicoides* is then estimated from the model that best fit the trap data in both experimental setups.

## Methods

Experiments were conducted in the summer of 2011, on a farm with approximately 70 cows in Klippinge, Denmark (geographical coordinates: N55.3619, E12.3234). The study field, measuring approximately 120 by 120 meters, was grazed by cattle during the day. Before dusk, the cattle were excluded from the field, but had access to enclosures on the western side of the study site. The surrounding land cover was grazed fields and grain fields, so there were no obstructions of *Culicoides* vision or flight next to the setup. Approximately 100 meters from the experimental setup there was a cow stable and a dunghill with potential breeding sites for the Obsoletus group. In a radius of 500 m, there were at least three ponds with potential breeding sites of the Pulicaris group. However, no breeding sites were monitored during the study period. The experiment was set up close to the cattle to ensure a high density of *Culicoides*. There were no other sources of light pollution present on the field during the experiments. *Culicoides* were caught using CDC 1212 mini light traps (www.johnwhock.com). These traps are equipped with a horizontally mounted 11 cm fluorescent tube emitting anisotropic UV-light. This means that the highest light-intensity is seen perpendicular to the tube and no light is seen from the ends of the tube. The light tubes were placed at a height of 180 cm and all light tubes were aligned along the transect line. Before each catch night, freshly charged batteries were installed on the traps. The starting time of sampling was decided each catch night to be when it was dark enough to perceive the light from the traps with the naked eye at a few meters distance. Traps were allowed to catch in intervals of one hour before they were emptied. Catch nights were chosen subjectively for optimal flight conditions for *Culicoides*: low wind speed; no precipitation; high air humidity; no fog in the air; and not too low temperature. Weather variables were monitored during the experiment using a Davis Vantage Pro 2 weather station. No farm animals were harmed during this study and permission to conduct the experiment was obtained from the land owners before the study.

### Experimental setup

#### Experimental setup A

**Figure 1:**
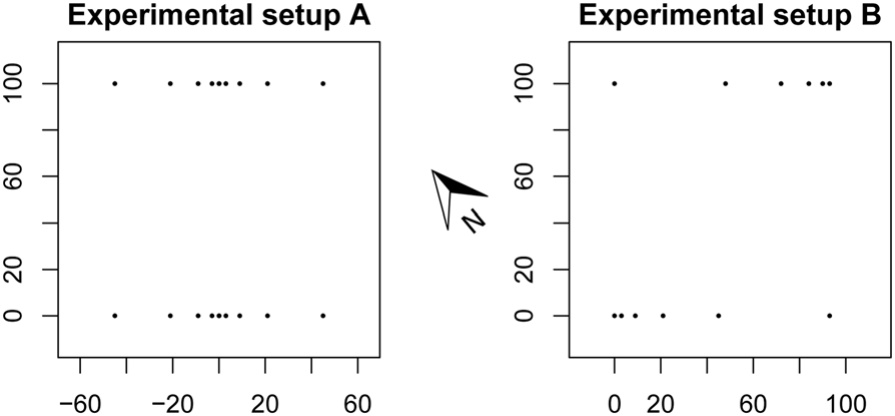
**Experimental setup.** Experimental setup A and B. The plots show the study area viewed from above. Distance units are meters. Traps are represented by black dots. All traps were hung with the light tubes along the transect line. In setup A, the middle dot represents two traps separated by 12 cm. In setup B, the configuration was rotated 90 degrees in some time intervals.

In the first experimental setup, 10 traps were set up in each of two transects with higher trap density towards the middle of the transects, aligned north-east to south-west (Figure 1). Within each transect, the traps were positioned at 0, 3, 9, 21 and 45 meters from the middle of the transect. In this way, the distance between traps was doubled for each transect position. In the middle position, two traps were placed with the light tubes separated by 12 cm. Two parallel transects were set up in each catch interval and separated by 100 meters. Setup A was run on the 27th (between 22.15 and 00.15 hours), 30th (21.15-00.15 hours) and 31st (22.15-00.15 hours) of July 2011.

#### Experimental setup B

In the second experimental setup, 6 traps were placed in two transects with higher density towards one end, also separated by 100 meters. This setup was either aligned north-east to south-west or north-east to south-west (Figure 1) in each catch interval. Within each transect, the traps were placed at 0, 3, 9, 21, 45 and 93 meters from the starting point, also doubling the distance between traps for each position. The transect directions were reversed so the end with more traps pointed in opposite directions. This setup was run on the 17th (N-S, 21.45-00.45 hours), the 18th (N-S, 21.45-00.45 hours) and on the 24th (E-W, 21.15-00.15 hours) of July 2011.

All light traps were equipped with new light tubes and were aligned so that all the tubes were parallel to the transect direction. Insects caught in the traps were sorted in a dissection microscope. *Culicoides* were identified by wing morphology according to Campbell & Pelham-Clinton [13] and female specimens of the *Culicoides obsoletus* group and the *C. pulicaris* group were identified and used for analysis. The two species groups contributed separately to the dataset so that the total number of study units were initially 54 transects, consisting of different species groups, hourly catch intervals and transect positions.

### Models

We investigated three mathematical models to explain the observed fraction of catch per trap per transect. Model I, ‘Nearest trap’, assumed a constant trap radius where vectors always fly to the nearest trap, as suggested by Rigot & Gilbert (2012) [11]. Model II, ‘Indirect light’, calculates the combined light field surrounding the traps, then assumes that *Culicoides* always fly toward areas of higher light intensity, and in this way range of attraction becomes defined by a cutoff value in total light intensity. Model III, ‘Perceived light’, assumes that *Culicoides* fly in the direction of what they perceive as the brightest light source. The model approximates the perception of a light trap for *Culicoides* with a Gaussian function, which means that the lights from closely placed traps overlap. Range of attraction is again defined by a cutoff in light intensity.

#### Model I - Nearest trap

**Figure 2:**
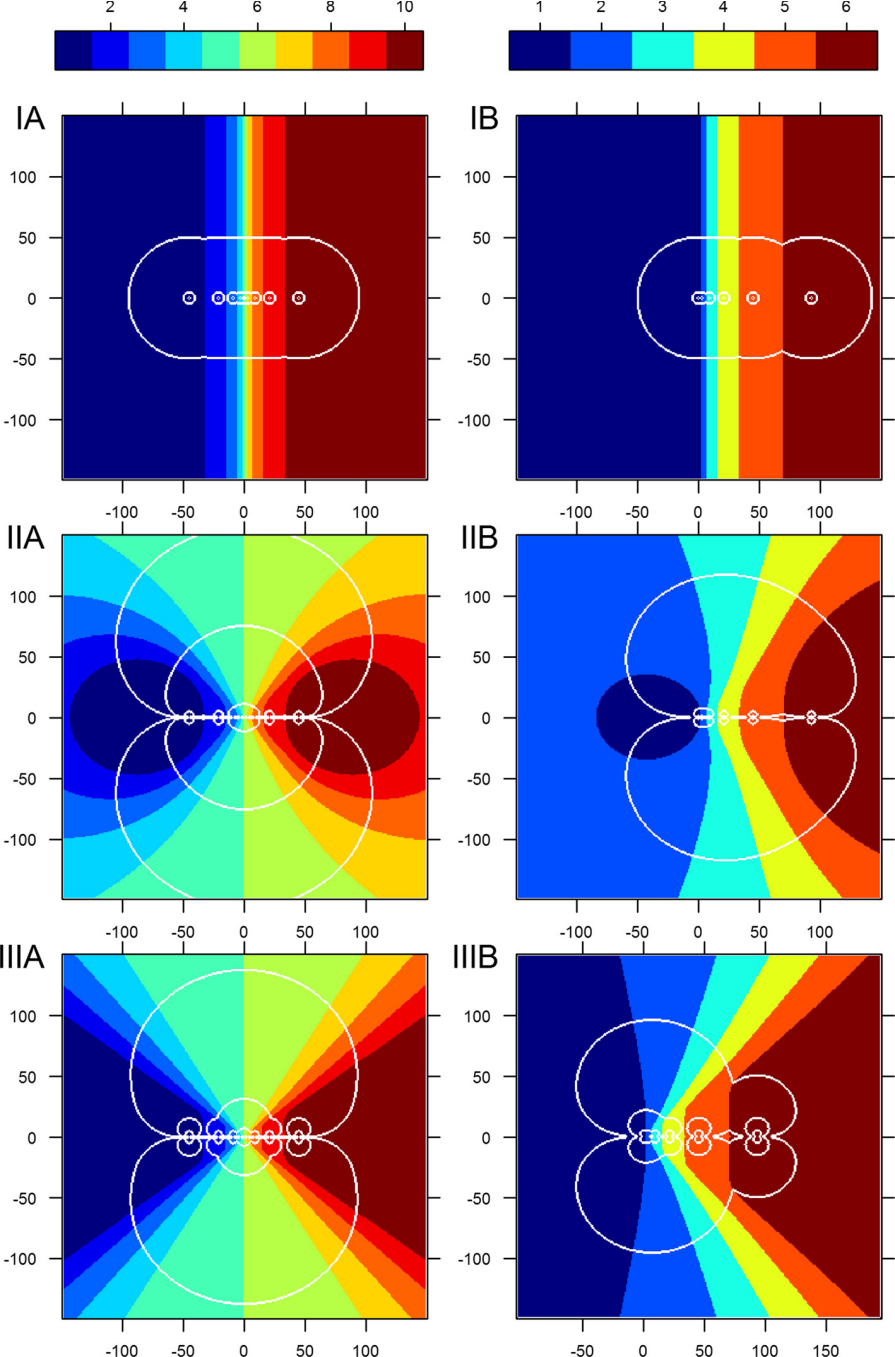
**Range of attraction vizualisation.** The area of attraction for the three models. It is assumed that each trap catches a number of *Culicoides* proportional to the area within the range of attraction (white lines). The plots represents modeled fields of 300 by 300 meters with one transect of traps from Figure 1. The colors (color-key on top) indicate which trap a *Culicoides* in this area will end up in, with trap numbers corresponding to numbers in Figure 5. The white lines indicate different cutoff points in range of attraction (light intensity). These plots thereby represent the functions *T*^*k*^(*x*,*y*), where the white lines in each plot indicate the three cutoffs, *I*_*C*_, also indicated in Figure 5, which corresponds to a range of attraction of 5 and 50 meters and the best fit. Model III is presented with *σ*=10. The left column is setup A, the right column is setup B. In model I (top) *Culicoides* always fly to the nearest trap, in model II (middle) *Culicoides* fly towards the area of highest light intensity, and in model III (bottom) *Culicoides* fly towards what they perceive as the brightest light, as illustrated in Figure 3.

Effectively this model states that *Culicoides* always fly towards the nearest trap. Consequently traps are assumed to catch a number of *Culicoides* proportional to the area within the range of attraction, *r* (IA/B in Figure 2). If the distance, *d*, between two neighboring traps is smaller than two times the range of attraction, each trap’s area of catch is reduced by half of the overlapping area. The model only allows for the catch area to be reduced by the nearest neighbor(s). The equations we present are only valid for traps on a line. The predicted fraction of catch, $C_{i}^{I}$, for the *i*’th trap is then:

(1) $$\begin{array}{lll} A_{i}^{I}\; & =\;\Pi r^{2}-\underset{j\in N\;N}{\sum }\left(2r^{2}\arctan\left(\frac{2\Omega }{d_{\text{ij}}}\right)-d_{\text{ij}}\Omega \right)\; & \;\\ \Omega \; & =\;\sqrt{r^{2}-{\left(\frac{d_{\text{ij}}}{2}\right)}^{2}}\; & \;\\ C_{i}^{I}\; & =\;\frac{A_{i}^{I}}{\overset{n}{\underset{i=1}{\sum }}A_{i}^{I}}\; \end{array}$$

**Figure 3:**
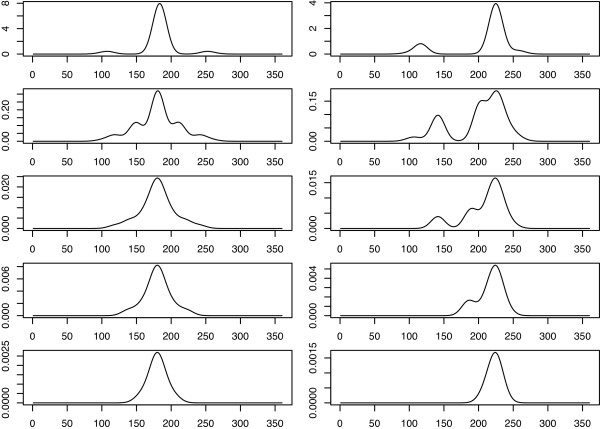
**360 degrees field of view of a *Culicoides*.** The light intensity of setup A as perceived by one *Culicoides* as a function of degrees on the angle of the transect. X-axes show the view angle in degrees where zero degrees is downwards orthogonal on the transect line and the angle increases counterclockwise. Plots show views as if a single *Culicoides* approaches the central traps in a straight line perpendicular to (left), or in a 45 degree angle to the transect (right). In the coordinate system of Figure 2 and equation (6), this corresponds to the coordinates (0,−*i*) (left) and (−*i*,−*i*) (right) where i = 1,5,25,50,100 meters, with *σ*=10. *Culicoides* are assumed to fly towards the brightest perceived light, and as the perception is assumed to be Gaussian, *Culicoides* must be close to the transect (distance depends on *σ*) to differentiate neighboring traps.

Where $A_{i}^{I}$ is the area within range of attraction for the *i*’th trap minus half the area of the *j*’th trap, which is the one or two next neighbors, *NN*, *d*_*ij*_ is the distance between the *i*’th and *j*’th trap, and *n* is the total number of traps in the transect. $A_{i}^{I}$ is represented by one color per trap (*i*) within the white lines (range of attraction) in Figure 2. This type of model was investigated for *Culicoides* by Rigot & Gilbert (2012) [11].

#### Model II - Indirect light

In this model we assume that *Culicoides* always fly toward areas of higher light intensity, quite similar to moving towards higher concentrations of scent molecules, when searching by smell. We therefore calculate the total light intensity, *I*(*x*,*y*), for the area around the traps:

(2) $$I(x,y)\;=\;\overset{n}{\underset{i=1}{\sum }}\frac{\Psi (\phi_{i}(x,y))}{{\left(x-x_{i}\right)}^{2}+{\left(y-y_{i}\right)}^{2}}$$

(3) $$\Psi (\phi_{i}(x,y))\;=\;|\cos(\phi_{i}(x,y))|$$

Where *n* is the number of traps and *x*_*i*_, *y*_*i*_ are the spatial coordinates of the *i*’th trap. *Culicoides* in any given position are then assumed to fly along the highest gradient of *I*(*x*,*y*). We model the anisotropic light field around traps using *Ψ*, which is a function of the angle around each trap, *ϕ*_*i*_(*x*,*y*), in concordance with Lambert’s cosine law. To model the attraction area we start one *Culicoides* for every square meter within at least 150 meters from the central trap, and simulate the attraction of light by individually moving them in small steps along the largest gradient in the surrounding light field until they eventually arrive at a trap location. From this we can determine a function *T*^*II*^(*x*,*y*) which tells which trap a single *Culicoides* will fly to as a function of initial position (IIA/B in Figure 2). We can then define a cutoff, *I*_*C*_, in light intensity that defines how far away *Culicoides* are attracted towards the light. And from this determine the fraction of catch of *Culicoides* for the *i*’th trap, $C_{i}^{I\;I}$:

(4) $$C_{i}^{I\;I}=\frac{\underset{x,y}{\sum }I\;(\;I(x,y)>I_{C}\wedge T^{I\;I}(x,y)=i)}{\underset{x,y}{\sum }I\;(\;I(x,y)>I_{C})}$$

Where **I** is the indicator function. This equation and Figure 2 is then interpreted as that each trap in the transect catch a fraction of *Culicoides* proportional to the area within the light cutoff.

#### Model III - Perceived light

This model aims to recreate the view that *Culicoides* have of the traps from every point on the area around the traps. *Culicoides* will fly in the direction of the perceived brightest light. For every point on the field a 360° view is generated with a resolution of 1°, *V*(*x*,*y*,*ϕ*). This is calculated by combining the light intensity from the *i*’th trap at every point, *I*_*i*_(*x*,*y*). Combined with anisotropic light, *Ψ*, and the inaccuracy *Culicoides* perceive the position of the light traps, *σ*.

(5) $$I_{i}(x,y)\;=\;\frac{\Psi (\phi_{i}(x,y))}{{\left(x-x_{i}\right)}^{2}+{\left(y-y_{i}\right)}^{2}}$$

(6) $$V(x,y,\phi )\;=\;\overset{n}{\underset{i=1}{\sum }}\frac{I_{i}(x,y)}{\sigma \sqrt{2\Pi }}\exp\left(-\frac{{\left(\phi -\phi_{i}(x,y)\right)}^{2}}{2\sigma^{2}}\right)$$

We again start one *Culicoides* for every square meter and simulate their flight towards what they perceive as the strongest light until they arrive at a trap location. From this the function *T*^*III*^(*x*,*y*) is determined which describes which trap a single *Culicoides* will fly to as a function of initial position (Figure 2). We define a cutoff, *I*_*C*_, in light intensity that defines how far away *Culicoides* are attracted towards the light. And from this we determine the fractional catch of *Culicoides* per transect:

(7) $$C_{i}^{I\;I\;I}=\frac{\underset{x,y}{\sum }I\;(\max(V(x,y,\phi ))>I_{C}\wedge T^{I\;I\;I}(x,y)=i)}{\underset{x,y}{\sum }I\;(\max(V(x,y,\phi ))>I_{C})}$$

The *Culicoides* fly in the direction they perceive to have the brightest light. When *Culicoides* are far away the trap lights seem to blend together because the angular distance between them is smaller and the *σ* smears the view. As illustrated in Figure 3.

##### Range of attraction

, *r*^*k*^, for model II and III must be calculated from the light intensity cutoff, $I_{C}^{k}$, which is a result from the best fit of models to data. In the experimental setups the combination of light from the traps provides a complex pattern for the combined range of attraction as seen in Figure 2. But for a single trap $I_{C}^{k}$ determines the range of attraction perpendicular to the tube as:

(8) $$\begin{array}{lll} r^{k}\; & =\;\sqrt{\frac{\kappa^{k}}{I_{C}^{k}}}\;\\ \kappa^{I\;I}\; & =\;1\;,\;\kappa^{I\;I\;I}=\frac{1}{\sigma \sqrt{2\Pi }}\; & \; \end{array}$$

With *κ*^*k*^ being the light intensity one meter from a trap in the *k*’th model. Model II and III are normalized differently because the total light intensity from one lamp across the view function, *V*, is set to sum to one in model III.

#### Fitting to data

To determine best fit we used the value of a *χ*^2^-test statistic (CS^*k*^) to evaluate the modeled fraction of catch $C_{i}^{k}$, from the *k*’th model, with the observed data, *C*_*i*,*j*_.

(9) $$\begin{array}{lll} {\text{CS}}^{k}\; & =\;\overset{n}{\underset{i=1}{\sum }}\overset{m}{\underset{j=1}{\sum }}\frac{{\left(C_{i,j}-E_{i,j}^{k}\right)}^{2}}{E_{i,j}^{k}}\;\\ E_{i,j}^{k}\; & =\;C_{i}^{k}(r^{k},\sigma )\times {\hat{\text{I}}}_{j}\; & \; \end{array}$$

Where *n* is the number of traps, *m* is the catch number with Î _*j*_ being an identity vector of length *j*, so $E_{i,j}^{k}$ is the expected fraction of catch from model *k* repeated *j* times equal to the number of separate catches. And $C_{i}^{k}$ is the fractional catch from the models, which is dependent on range of attraction and also *σ* in model III. The best fit is the set of parameters (*r*^*k*^,*σ*) that minimizes the value of CS^*k*^. This method puts equal weight on each transect of catch. Given that the *C*_*i*,*j*_ is the relative catch per trap per transect, the abundance of *Culicoides* does not have any impact on the analysis. This removes the need to include factors which affect abundance in the model.

All of the data was not included in the trap data. A transect of trap data was omitted if there were more than three zero catches, which was usually observed on days with a very low total catch. Many zero catches would bias the catch distribution towards equal catches between traps, which would not represent the true catch distribution, but merely reflect the variation in daily catch. In total 14 of 28 trap data sets for setup A were excluded, while only 5 of 26 where omitted for setup B. Thus a total of 35 transects were included in the analysis. The analysed data from setup A was consequently from the 27th (between 23.15 and 00.15 hours), the 30th (23.15-00.15 hours) and the 31st (22.15-00.15 hours) of July 2011. For setup B analysed data was from the 17th (N-S, 23.45-00.45 hours), the 18th (N-S, 21.45-00.45 hours) and the 24th (E-W, 22.15-00.15 hours) of July 2011.

In the data there were three missing data points (NAs) for setup A and two NAs for setup B. These were handled by using an Expectation Maximization (EM) procedure [14]. The EM converged in all cases after a maximum of one step.

In setup A data is presented symmetrized by averaging over traps in pairs around the center of one transect of traps. We symmetrized data to remove directional bias in the experimental setup. However, the symmetrizing did not affect fitting with the CS function, and symmetrized and un-symmetrized data gave the same results. We chose to present the data symmetrized to allow for a better visual comparison with the models, which will always give a symmetrical result for setup A.

Confidence intervals on *r* and *σ* in model III were determined using Fischer information theory as presented in Madsen (2008) [15]. The method was implemented by approximating the CS-test curve to a second order polynomial using a power transformation to symmetrize around the minimum value of the CS function (9). Notice that this method produces non-symmetric confidence intervals. To ensure that the model was not driven by the catch on one night, one experimental type of setup, or one *Culicoides* species we used the jackknife method on the data. Which is to reanalyze the data excluding the data from one catch night at the time, each species group, or each experimental setup at the time.

## Results

10,150 *Culicoides* were caught and included in the analysis, of which 1,817 specimens were from the *C. obsoletus* group and 8,333 specimens were from the *C. pulicaris* group. The hourly catches from each transect ranged between 3.6-27.8 (mean: 13.0) specimens per trap for the *C. obsoletus* group and 2.8-177.8 (mean: 52.5) specimens per trap for the *C. pulicaris* group. Each transect in setup A comprised 90-278 (mean: 179.1) specimens from the *C. obsoletus* group and 312-1778 (mean: 970.3) specimens from the *C. pulicaris* group. In setup B, each transect comprised 22-144 (mean: 61.8) specimens from the *C. obsoletus* group and 17-112 (mean: 63.4) specimens from the *C. pulicaris* group.

The catch nights were chosen subjectively for optimal flight conditions for *Culicoides*. During the catches, the temperature was between 12.9 and 18.6 degrees Celcius. The dew point temperature was below the ambient temperature during the whole study, and the air humidity was between 72% and 94%. The wind speed was between 0 and 1.8 m/s, and no precipitation was measured. Thus there was no rain, no fog in the air, high humidity, low wind speed and not too low temperatures.

**Figure 4:**
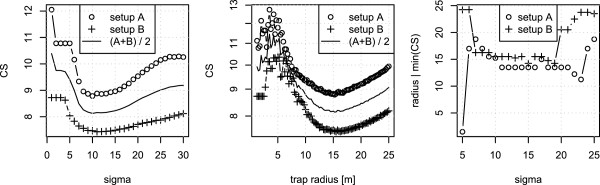
**Model fits of sigma and range of attraction.** CS values from eq. (9) as a function of *σ* (left), and range of attraction, *r*, with *σ*=10 (middle). The minimum CS value indicates which value best fits with the observed data, when using model III. Right is the range of attraction, *r*, that minimizes CS for different values of *σ*, which shows a stable range of attraction for a range of *σ*. All plots are for model III. The jump in values around *σ*=20 in the figure furthest to the right is due to range of attraction exceeding the simulation box size. The noise for small trap radius and small *σ*s is due to rounding errors that occur on small scales.

The best fit of the models was determined by minimizing (9) as a function of the range of attraction *r* for model I, as a function of light intensity cutoff *I*_*C*_ in model II, and as a function of light intensity cutoff *I*_*C*_ and *σ* in model III (Figure 4). For model II and III, *I*_*C*_ is recalculated to *r* by using (8).

**Figure 5:**
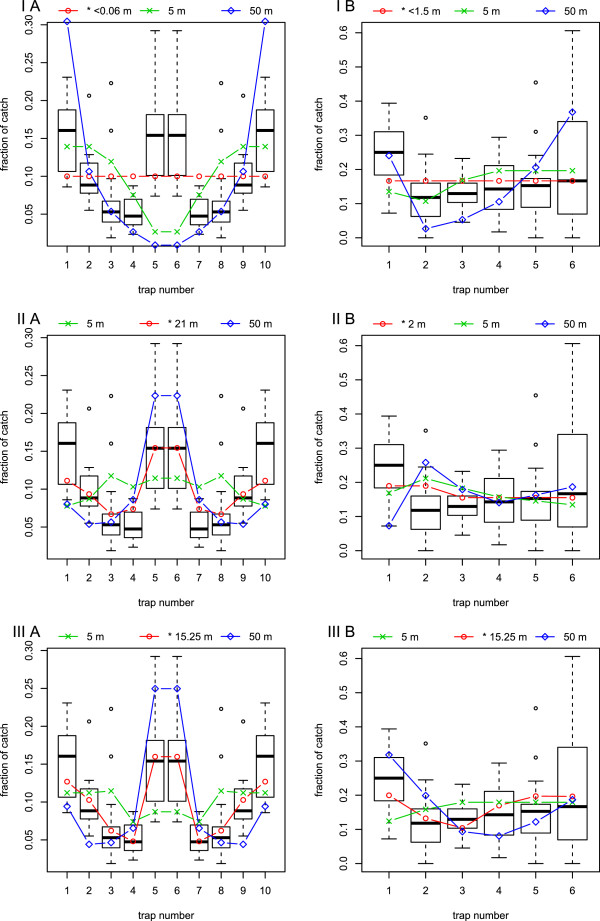
**Model fits to experimental setups.** Predicted fractional catch per trap given model I (nearest trap), II (indirect light), and III (perceived light) (top, middle, bottom) with the field data as boxplots, for setup A and B (left, right). The prediction is the fractional part of the area covered within the range of attraction in Figure 2. The red line with circles is always the best fit of the model to the data. The predicted green and blue lines is fractional catch with a range of attraction of 5 or 50 meters. The ranges of attraction *r*^*k*^ giving the best fit for the *k*’th model were: *r*^*IA*/*B*^<0.06/1.5 m, *r*^*IIA*/*B*^=21/<2 m, and *r*^*III*^=15.25 m with *σ*=10.

The catch distribution of different ranges of attraction and the best fits of the models are presented in Figure 5. The collected trap data are numbered as presented in Figure 1. from left to right at *y*=0. We generally observed that traps catch a lower fraction of *Culicoides* when placed closer together. However, there are two very clear exceptions when traps are placed close together. The two central traps in setup A (number 5 and 6) catch almost the same as the outermost traps, and trap 1 in setup B caught a higher fraction than the other traps. These observations are strong indications that closely placed traps do not only compete for *Culicoides*, but also amplify attraction. The characteristics of the models compared to data are as follow (as seen in Figure 5).

Model I - Nearest trap: When range of attraction is lower than half the distance between the closest traps, the traps do not compete and will catch the same fraction of *Culicoides*. When going towards higher trap radius the center traps in setup A will catch a lower and lower fraction due to competition with neighboring traps. The outermost traps will always catch the highest fraction due to the lowest competition. Model I is therefore unable to reproduce the remarkable peak in the middle traps which is observed in data from experimental setup A. Moreover, it overestimates the catch in the outermost traps in setup B.

Model II - Indirect light: In this model *Culicoides* are attracted towards higher total light intensity, which is a very simple way of considering attraction to light. Flying towards the strongest concentration of light attracts more *Culicoides* to the central area of the trap setups. This explains that for setup B the model predicts that traps 2 and 3 for medium and large ranges of attraction catch more than the outermost traps, which is contrary to the observed data. We therefore observe that the fitted *r*^*II*^ is very different whether fitted to setup A or B.

Model III - Perceived light: *Culicoides* in this model will fly directly towards the perceived brightest light source. The two central traps in setup A are located within such a small distance that *Culicoides* cannot distinguish them until within a very short distance, therefore they will appear as one trap with twice the brightness (Figure 3). This gives the added attraction to the middle traps which produce the central spike in the trap catch distribution, which is also observed in data from setup A. Model III also fits data setup B where competition among traps 2, 3, and 4 reduce their fraction of catch compared to the outermost traps. Moreover, trap 1 is predicted to catch a higher fraction due to to the nearness of trap 2.

In model III the CS function for both setup A and setup B displayed a global minimum at *σ*=10 (left in Figure 4). With a combined 95% confidence interval 7.2-13.8. For *σ*=10 the CS is a continuous function of *r* with a unique global minimum at *r*^*III*^=15.25 meters for both setup A and B (middle in Figure 4). With a 95% confidence interval 12.7-18.3. Furthermore, we observed that for a broad range of *σ*s (also covering the 95% confidence interval) the optimal single range of attraction is approximately the same for setup A and B (right in Figure 4). Even though *r*^*III*^ is reported to 15.25 meters please note that CS values were only calculated per one quarter of a meter, and the precision is not 1 centimeter.

The jackknife tests indicated that the range of attraction did not change significantly when excluding any of the catch nights, experimental setups, or species groups. In model III the only significant aberration of the value of *σ* was when excluding the catches on 30.07.11, on this date the estimate changed to 14 with a 95% confidence interval 9.9-19.7, while all other results where within confidence levels (data not shown).

We notice that model I does not fit any of the data (Figure 5, top). Model II can only fit data with very different values for *r* (Figure 5, middle). While model III is able to fit both setup A and B using the same values for *σ* and *r* (Figure 5, bottom). Since Model III is the only model which can fit both experimental setups with the same values of *r* we have not included a comparison of models using information criteria.

## Discussion

In this study we found a range of attraction for *Culicoides* at 15.25m. This means that the trap type used in this study should be separated by at least 30.5m (25.4-36.6) to enable independent sampling. When traps are placed closer than this, they will influence each other, competing for *Culicoides*. However, the range of attraction will also be extended when catch areas overlap, which is a novel result of this study. Thus, it is possible to cover a greater area from the same position by using more than one trap.

We used the relative levels of catches to estimate the range of attraction. This made the modelling independent of weather parameters causing changes in the abundance, e.g. wind speed or temperature. Spatial parameters, e.g. wind direction and location of hosts are likely to have an impact on the relative catches in the traps. However, we made an effort to compensate for this in the symmetrical shape of setup A, reversing the transects in setup B and by rotating setup B (Figure 1).

The range of attraction may differ between species. But Rigot *et al.* (2012) [11] found very similar ranges of attraction between vector species and vector species groups with overlapping confidence intervals. Because some species (e.g. *C. impunctatus*) may not be attracted by light as much as others [16], the range of attraction may be different for different species. Therefore, we conducted a jackknife test on the results by removing a species group one at a time, which showed that the results did not differ significantly when testing the species groups individually.

Trap efficiency is dependent on the background illumination, which can differ between sampling periods due to factors such as cloudiness, moon phase and time of sampling related to sunset (e.g. [17-21]). This is a potential source of bias and could result in different ranges of attraction between sampling periods. However, we tested this in a jackknife analysis, leaving out one catch night at a time, and found no significant difference in the estimate of the range of attraction. The background illumination is also more likely to impact on the *σ* parameter in model III because we expect that the *Culicoides* can distinguish light sources better under darker conditions. As previously stated, leaving out one of the catch nights did yield a significantly different estimate of *σ* (data not shown).

Model I (Nearest trap) failed to fit the data in experimental setup A and B. This model has a fixed range of attraction for each trap regardless of the distance to neighboring traps. This model type was used in the study of Bidlingmayer & Hem (1980) [18] to explain catch patterns of mosquitoes in traps without light, and it was recently used in another study to fit catches of *Culicoides* in light traps [11]. Given the physical properties of light, the effect of two neighboring light sources create an additive effect in the overlapping area, a main assumption in model II and model III. Thus, we can see from this study that the range of attraction from one point can be extended by using more traps, corresponding to a stronger light source.

Model III (Perceived light) was the only model to fit both experimental setup A and B (Figure 5). Thus, we regard 15.25m (12.7m-18.3m) as a reliable estimate of the range of attraction for one trap for *Culicoides* vectors. Rigot & Gilbert (2012) [11] found that the 8W Onderstepoort type traps had a range of attraction of 29.6 (26.3m-31.9m). However, since the model used in that study failed to fit both experimental setups in our study, a more precise estimate may be obtained by using Model III from the present study. The fact that Venter *et al.* (2012) [12] found a range of attraction between 2 and 4 meters for the Onderstepoort type trap, could indicate that other unknown factors may be important when traps are allowed to catch the whole night through.

As stated in [22], the range of attraction covers three concepts: the distance at which specimens can physically reach the trap within a given time interval; the distance at which a specimen can detect the trap; and the distance at which a specimen shows directed movement towards the trap. If traps are allowed to sample a longer time, data can be influenced by other parameters such as wind direction and wind plumes created by host animals. If sampling time is too short, the specimens within the range of attraction may not be able to reach the trap before the sampling ends. To investigate the range of attraction and the influence of time, different sampling intervals would be needed, which is worth further research.

The distance from the *Culicoides* to a trap is also worth considering. We assumed general random flight with full attraction towards the traps within the range of attraction. However, the traps might attract a higher percentage of *Culicoides* in the vicinity closer to the traps compared to further out in the range of attraction, possibly proportional to light intensity.

In our models we assumed that *Culicoides* disperse evenly within the field. However, the abundance of *Culicoides* is likely to be higher near the cattle. We have tried to compensate for this by reversing the direction of the transects in setup B. Setup A compensates for this by the symmetry of the transect (Figure 1). Furthermore, the *Culicoides* may not be evenly dispersed when consecutive trapping is carried out because *Culicoides* within the range of attraction would be caught in the first trapping period and thus new *Culicoides* in the area would have to migrate in by random flight. To explain this pattern, a better fit may be obtained by fitting data to the circumference of the attraction area rather than the area itself. This could be worth investigating in future research. In both model II and model III, we assumed that all *Culicoides* caught in the traps approached the traps from the same height as the light sources, and therefore there were blind angles in the ends of the light tubes. If the *Culicoides* approached the traps from a lower height, the blind angle would be less pronounced. Although *Culicoides* have been caught at higher altitudes (e.g. [23]), and Venter *et al.* (2009) [24] caught most *Culicoides* at a height of 2.8m in South Africa, the main flying height for *Culicoides* vectors in northern Europe is still unknown.

In Models II and III the light distribution around each trap was anisotropic. We also simulated these two models using isotropic light, but for both models anisotropic light fitted data better (data not shown). This indicates that the direction of the light tube in the trap is important, which has practical implications when catching *Culicoides*. If a certain area is to be monitored in a study, e.g. an enclosure with host animals, the catch size will depend on the angle of the trap to the area of interest. If trap catches are to be compared in a study, the standardization procedure should include direction of the light traps.

In this study, Model II modelled the light from each trap, resulting in higher light intensity when ranges of attraction overlap. This is comparable to a scent zone created isotropically around a host animal if there is no wind present. This model did not fit data as well as Model III did, where the *Culicoides* can perceive the individual sources of light at a distance and head for the strongest light source. This is an important biological finding and indicates that the *Culicoides* show directed movement towards a light source rather than a more random flight towards areas with higher light intensity. The implications of this finding is important for other studies using light trap catches to estimate the number of *Culicoides* (and possibly also other insects, e.g. mosquitoes) attracted to host animals. We have shown here that the vectors evaluate light sources at a distance. This behaviour is different from how we assume the vectors are attracted to host animals, i.e. following a plume of scent. Thus light trap catches may not represent the number and species of vectors attracted to hosts very well, and should be used with caution.

## Conclusions

We tested three different models to fit two different field data sets, and showed that the *Culicoides* are likely to locate the trap by evaluating the direction of the strongest light source in their field of view and then fly towards it rather than flying towards the nearest trap. We estimated the range of attraction for a single CDC 4W UV light trap to be 15.25m (12.7-18.3) perpendicular to the light tube. Therefore, we suggest that, in future studies, traps of this type are separated by at least 30.5m (25.4-36.6) in order to be independent. If they are placed closer than this, their interactions should be modelled as in model III in this study. Light traps may not represent the number of vectors attracted to hosts because the attraction behaviours are different.

## Competing interest

The authors declare that they have no competing interests.

## Authors’ contributions

This project is a part of the PhD project by CK at the Veterinary Institute at the Technical University of Denmark and the PhD project by KG at the Veterinary Institute and Department for Informatics and Mathematical Modelling, Technical University of Denmark. CK conceived the study, carried out the field work, participated in discussion of the modeling procedure and wrote the background, experimental setup, experimental setup results, discussion and conclusion sections of the manuscript and created Figure 1. KG conceived the framework of models II and III, carried out all modeling and model fitting and wrote the modeling and the model results sections of this paper. KG also participated in the discussion and conclusions sections and created Figures 2, 3, 4 & 5. RB participated in conceiving the study, planning of field experiments and discussion of the results. AS participated in the planning of the field work and model fitting and evaluation. LEC participated in conceiving model III, the modeling procedure, model fitting and evaluation. All authors read and approved the final version of the manuscript.

## References

[B1] Carpenter S Lunt H Arav D Venter G Mellor P Oral susceptibility to bluetongue virus of Culicoides (Diptera: Ceratopogonidae) from the United Kingdom J Med Entomol 2006 43 73 78 10.1603/0022-2585(2006)043[0073:OSTBVO]2.0.CO;2 16506450

[B2] Rasmussen L Kristensen B Kirkeby C Rasmussen T Belsham G Bødker R Bøtner A Culicoids as vectors of schmallenberg virus Emerg Infect Dis 2012 18 7 1204 1206 22709978 10.3201/eid1807.120385 PMC3376822

[B3] Venter G Labuschagne K Hermanides K Boikanyo S Majatladi D Morey L Comparison of the efficiency of five suction light traps under field conditions in South Africa for the collection of Culicoides species Vet Parasitol 2009 166 3 299 307 10.1016/j.vetpar.2009.08.020 19758757

[B4] Allan S Day J Edman J Visual ecology of biting flies Annu Rev Entomol 1987 32 297 314 10.1146/annurev.en.32.010187.001501 2880551

[B5] Purse B Tatem A Caracappa S Rogers D Mellor P Baylis M Torina A Modelling the distributions of Culicoides bluetongue virus vectors in Sicily in relation to satellite-derived climate variables Med Vet Entomol 2004 18 2 90 101 10.1111/j.0269-283X.2004.00492.x 15189233

[B6] Conte A Goffredo M Ippoliti C Meiswinkel R Influence of biotic and abiotic factors on the distribution and abundance of Culicoides imicola and the Obsoletus Complex in Italy Vet Parasitol 2007 150 4 333 344 10.1016/j.vetpar.2007.09.021 17997043

[B7] Takken W Verhulst N Scholte E Jacobs F Jongema Y van Lammeren R The phenology and population dynamics of Culicoides spp. in different ecosystems in The Netherlands Prev Vet Med 2008 87 41 54 10.1016/j.prevetmed.2008.06.015 18639947

[B8] Odetoyinbo J Preliminary investigation on the use of a light-trap for sampling malaria vectors in the Gambia Bull World Health Org 1969 40 4 547 5306720 PMC2556112

[B9] Baker R Sadovy Y The distance and nature of the light-trap response of moths Nature 1978 276 818 821 10.1038/276818a0

[B10] Truxa C Fiedler K Attraction to light–from how far do moths (Lepidoptera) return to weak artificial sources of light? Eur J Entomol 2012 109 77 84

[B11] Rigot T Gilbert M Quantifying the spatial dependence of Culicoides midge samples collected by Onderstepoort-type blacklight traps: an experimental approach to infer the range of attraction of light traps Med Vet Entomol 2011 26 2 152 161 22098421 10.1111/j.1365-2915.2011.00988.x

[B12] Venter G Majatladi D Labuschagne K Boikanyo S Morey L The attraction range of the Onderstepoort 220V light trap for Culicoides biting midges as determined under South African field conditions Vet Parasitol 2012 190 1-2 222 229 10.1016/j.vetpar.2012.05.019 22704896

[B13] Campbell J Pelham-Clinton E X.A taxonomic review of the british species of Culicoides Latreille (Diptera, Ceratopogonidæ) Proc R Soc Edinburgh Sect B Biol 1960 67 03 181 302 10.1017/S0080455X00000758

[B14] Little R Rubin D Statistical Analysis with Missing Data 1987 New York: Wiley

[B15] Madsen H Time Series Analysis 2008 CRC Press

[B16] Holmes P Boorman J Light and suction trap catches of Culicoides midges in Southern England Med Vet Entomol 1987 1 4 349 359 10.1111/j.1365-2915.1987.tb00366.x 2979552

[B17] Bowden J Church B The influence of moonlight on catches of insects in light-traps in Africa. Part II. The effect of moon phase on light-trap catches Bull Entomol Res 1973 63 01 129 142 10.1017/S0007485300050938

[B18] Bidlingmayer W Hem D The range of visual attraction and the effect of competitive visual attractants upon mosquito (Diptera: Culicidae) flight Bull Entomol Res 1980 70 02 321 342 10.1017/S0007485300007604

[B19] Muirhead-Thomson R Trap Responses of Flying Insects. The Influence of Trap Design on Capture Efficiency 1991 Academic Press Limited

[B20] Nowinszky L Nocturnal illumination and night flying insects Appl Ecol Environ Res 2004 2 17 52

[B21] Silver J Mosquito Ecology: Field Sampling Methods 2007 Springer Verlag

[B22] Wall C Perry J Range of action of moth sex-attractant sources Entomologia experimentalis et applicata 1987 44 5 14 10.1111/j.1570-7458.1987.tb02232.x

[B23] Chapman J Reynolds D Smith A Smith E Woiwod I An aerial netting study of insects migrating at high altitude over England Bull Entomol Res, London 2004 94 2 123 136 10.1079/BER2004287 15153295

[B24] Venter G Hermanides K Boikanyo S Majatladi D Morey L The effect of light trap height on the numbers of Culicoides midges collected under field conditions in South Africa Vet Parasitol 2009 166 3 343 345 10.1016/j.vetpar.2009.09.003 19800737

